# Cost-effectiveness analysis of mHealth applications for depression in Germany using a Markov cohort simulation

**DOI:** 10.1038/s41746-024-01324-0

**Published:** 2024-11-17

**Authors:** Bettina Freitag, Marie Uncovska, Sven Meister, Christian Prinz, Leonard Fehring

**Affiliations:** 1 Health Care Informatics, Faculty of Health, School of Medicine, Witten/Herdecke University, Alfred-Herrhausen-Straße 50, 58455 Witten, Germany; 2 Department Healthcare, Fraunhofer Institute for Software and Systems Engineering, Emil-Figge-Straße 91, 44227 Dortmund, Germany; 3 Faculty of Health, School of Medicine, Witten/Herdecke University, Alfred-Herrhausen-Straße 50, 58455 Witten, Germany; 4 Helios University Hospital Wuppertal, Medizinische Klinik 2, Heusnerstraße 40, 42283 Wuppertal, Germany

**Keywords:** Health care, Health care economics, Health policy

## Abstract

Regulated mobile health applications are called digital health applications (“DiGA”) in Germany. To qualify for reimbursement by statutory health insurance companies, DiGA have to prove positive care effects in scientific studies. Since the empirical exploration of DiGA cost-effectiveness remains largely uncharted, this study pioneers the methodology of cohort-based state-transition Markov models to evaluate DiGA for depression. As health states, we define mild, moderate, severe depression, remission and death. Comparing a future scenario where 50% of patients receive supplementary DiGA access with the current standard of care reveals a gain of 0.02 quality-adjusted life years (QALYs) per patient, which comes at additional direct costs of ~1536 EUR per patient over a five-year timeframe. Influencing factors determining DiGA cost-effectiveness are the DiGA cost structure and individual DiGA effectiveness. Under Germany’s existing cost structure, DiGA for depression are yet to demonstrate the ability to generate overall savings in healthcare expenditures.

## Introduction

Worldwide 970 million people suffer from mental illness^1,2^, whereof depression is with around 350 million one major diagnosis^1,3–5^. The COVID-19 pandemic led to a further increase of depression cases^6^. Consequently, mental illnesses are the third most common illness causing absence days at work^7^, with 30.4% of absence days caused by depressive episodes^8^. At the same time the access to therapy (especially psychotherapy) is limited and waiting times are high - according to estimates by the World Health Organization, only one in four affected people receives adequate treatment^4^. With increased disease pressure on the one hand and limited treatment capacities on the other, mobile health applications are discussed as new therapeutic option to improve patients’ access to care and generate positive health effects^9–14^. A meta-analysis of the effect of depression mobile health applications showed a reduction of depressive symptoms of patients using depression mobile health applications^15^. Germany was the first country worldwide to allow prescription of certified mobile health applications by physicians with cost coverage by statutory health insurances. These apps are called “Digitale Gesundheitsanwendungen” (DiGA) or in English “digital health applications”. Existing research shows that DiGA have significant potential to facilitate patient access to health care and are a valuable complement to existing therapies^16–21^. To gain prescribing and reimbursement status, DiGA must undergo a comprehensive certification process and provide scientific evidence of effectiveness through clinical trials and compliance with general requirements (e.g., data protection, safety, interoperability)^22^. In Germany, 42% of all DiGA are developed and approved for mental illnesses, whereof 24% address depression^23^. These apps offer functionalities such as cognitive-behavioral therapy exercises, mood tracking and psychoeducation in order to provide personalized and accessible mental health care to patients. The effectiveness of DiGA for depression have been demonstrated through clinical studies showing significant improvements in depressive symptoms (e.g., effect size of 1.63 of changes of the Beck Depression Inventory II of one exemplary depression DiGA^24^). In addition to this, DiGA aim to increase patient engagement and enhance adherence to treatment plans. DiGA are not intended to replace standard therapy, but to support it or offer a bridge to in-person therapy^23,25^. A study comparing the effectiveness of different internet-based interventions for depression in Germany came to the conclusion that there is a possible superiority of the interventions listed in the DiGA directory compared to other freely available internet interventions^26^. While the required medical studies mainly focus on the evaluation of the treatment efficacy of DiGA, little is known about the cost-effectiveness of DiGA for the healthcare system^21^. The development of standard methods and evaluation criteria for the economic benefits of mobile health applications and services in general is challenging due to the great amount of different applications and valuation approaches^27–29^. As current economic valuation lacks robustness and overarching comparability^30^, the development of an aggregated value function for assessing the benefits and costs to understand the value of care is still an unaddressed research field^27,31–33^. Past research developed a pragmatic patient centered framework to assess the economic value of medical evidence of mobile health applications in the United States and the United Kingdom focusing on improvement of quality adjusted life years (QALY)^34^. A Markov simulation model was previously used to evaluate the cost-effectiveness of mobile health-based integrated care for atrial fibrillation in China^35^. Another study examined the cost-effectiveness of a DiGA for patients with low back pain in Germany using a state-transition Markov model^36^. As part of an initiative of the European Commission a “Monitoring and Assessment Framework for the European Innovation Partnership on Active and Healthy Ageing (MAFEIP)” was developed^37^. This web-based tool allows a comparative assessment of a specific health technology to a standard of care scenario and is based on a Marcov model with three health states in its initial version^38^.

We aim to develop a methodological approach to financially evaluate the cost-effectiveness of mobile health applications for depression and to provide a calculation and interpretation example for the German DiGA market. Our design criteria were focused on creating a mathematical model that is easily comprehensible and usable by a wide range of researchers and analysts. Thereby we focused on a health insurance perspective and differentiated between different severity levels of depression, namely mild, moderate and severe depression.

## Results

### Simulation result of the three treatment scenarios

**Fig. 1:**
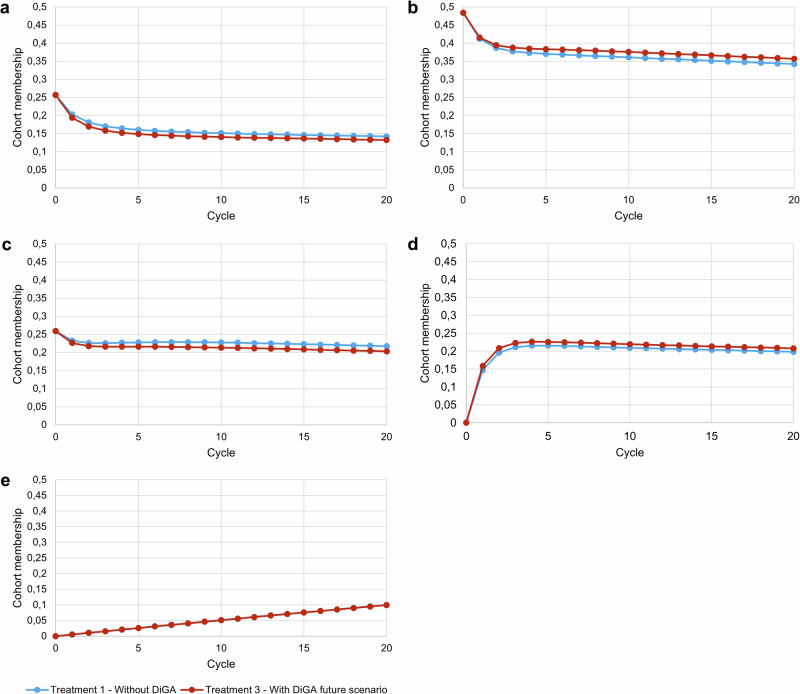
Markov probability analysis. Treatment 2 is excluded from the visualization since the value differences to treatment 1 are too marginal to be recognized in the graph. Cycle **=** 0 represents the starting proportions, cycle **=** 20 shows the proportions at the end of the simulation horizon. One cycle length is equal to 3 months. **a** Shows the mild depression health state, **b** Shows the moderate depression health state, **c** shows the severe depression health state, **d** shows the remission health state and **e** shows the death health state. Blue line = Treatment 1 without DiGA; red line = Treatment 3 with DiGA future scenario. The Markov probability analysis graphs show the proportions of the cohort in treatment 1 and treatment 3 belonging to the defined health states over the simulation horizon.

**Fig. 2:**
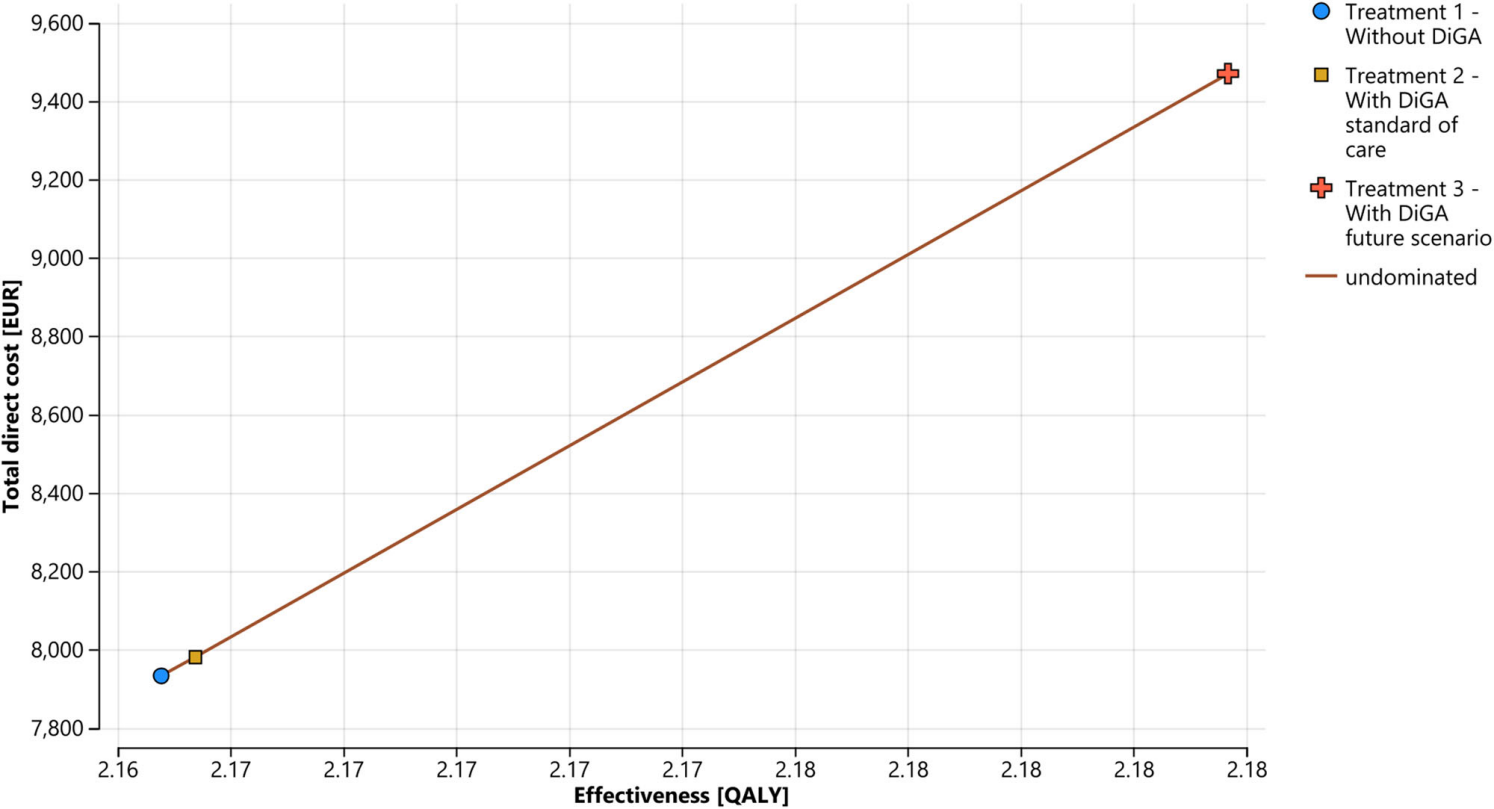
Cost-effectiveness plane. The graph shows the effectiveness and total direct costs of each treatment scenario over the simulation horizon and per patient. Blue dot = Treatment 1 without DiGA; yellow square = Treatment 2 with DiGA standard of care; red cross = Treatment 3 with DiGA future scenario.

Figure 1a–e shows the development of the patient cohort membership over the simulation horizon for each health state. It can be observed that DiGA treatment effectively delays disease progression as demonstrated by a larger proportion of the cohort remaining in the remission health state and a smaller proportion of the cohort in the severe depression health state. Over the 5 year simulation horizon, the total direct costs of care with DiGA (treatment 2 / 3) and care without DiGA were ~ 39.720 / ~ 47.130 billion EUR and ~ 39.482 billion EUR respectively for the defined patient cohort. Based on our deterministic input data, in total treatment with DiGA is more expensive. The QALY gain was 10.777 / 10.869 million for DiGA treatment. Comparing the future DiGA scenario with the treatment without DiGA, DiGA treatment gained additional QALYs of ~ 0.02 per patient and 94,029 in total for the cohort with an incurred total direct costs of 1536 EUR per patient and ~ 7.648 billion EUR in total over the simulation horizon (In comparison, a study evaluating the cost-effectiveness of enhanced access to psychological therapies found a QALY gain of 0.014^39^. Another study, which compared cognitive-behavioral therapy with standard care, reported a QALY gain of 0.053^40^). Table 1 summarizes the results and Fig. 2 shows the cost-effectiveness plane of the three defined treatment strategies. One can observe that neither treatment scenario 2 nor treatment scenario 3 dominates treatment scenario 1. The INMB analysis shows negative values in both DiGA treatment strategies. A negative INMB value indicates that the incremental health effects of an intervention do not outweigh its cost at a given WTP threshold value compared to an alternative intervention^41^. The ICER analysis shows a higher value than the WTP threshold value of 54,794 EUR for treatment scenario 2 and 3. Consequently, according to the ICER analysis, both DiGA treatment scenarios would not represent a cost-effective treatment alternative compared to treatment scenario 1 without DiGA. Considering a pure economic point of view, treatment scenario 1 would be the option of choice in the base case result given the current cost structure in Germany.

**Table 1:** Simulation result using deterministic input variables (simulation horizon of 5 years)

Treatment scenario	Cumulated total direct costs base case [in EUR]		Cumulated effectiveness base case [in QALY]		Incremental net monetary benefit (INMB), comparator: Treatment 1 [in EUR]	Incremental cost effectiveness ratio (ICER), comparator: Treatment 1 [EUR/QALY]	Ranking treatment scenarios
	*Per patient*	*Per cohort*	*Per patient*	*Per cohort*			
Treatment 1: Without DiGA scenario	7,933	~39,482 million	2.16478	10,774,482			1
Treatment 2: With DiGA scenario standard of care	7,980	~39,720 million	2.16538	10,777,473	-14.84	79,466	2
Treatment 3: With DiGA future scenario	9,469	~47,130 million	2.18367	10,868,511	-501.43	81,335	3

### Sensitivity analysis

**Fig. 3:**
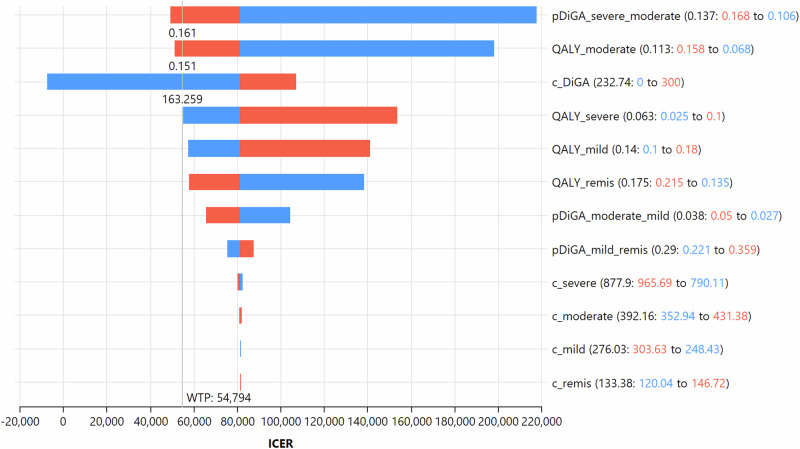
Tornado diagram of one-way sensitivity analysis assessing the effect of selected parameters on the ICER. ICER = Incremental cost effectiveness ratio, WTP = willingness-to-pay ratio referred to quality-adjusted life years; the horizontal bars represent the range of ICER due to changes in the model’s input parameters for one average patient in the cohort. Codification in the legend according to the following scheme: Input variable name (base case value: upper/lower range to lower/upper range), red = upper range of the input variable variation; blue = lower range of the input variable variation. p_DiGA_severe_moderate = transition probability to move from severe to moderate depression health state with use of DiGA; QALY_moderate = quarterly quality adjusted life years of an average patient in moderate depression health state; c_DiGA = quarterly cost of using a DiGA; QALY_severe = quarterly quality adjusted life years of an average patient in severe depression health state; QALY_mild = quarterly quality adjusted life years of an average patient in mild depression health state; QALY_remis = quarterly quality adjusted life years of an average patient in remission health state; pDiGA_moderate_mild = transition probability to move from moderate to mild depression health state with use of DiGA; pDiGA_mild_remis = transition probability to move from mild depression to remission health state with use of DiGA; c_severe = quarterly total direct costs of an average patient in severe depression health state; c_moderate = quarterly total direct costs of an average patient in moderate depression health state; c_mild = quarterly total direct costs of an average patient in mild depression health state; c_remis = quarterly total direct costs of an average patient in remission health state.

**Fig. 4:**
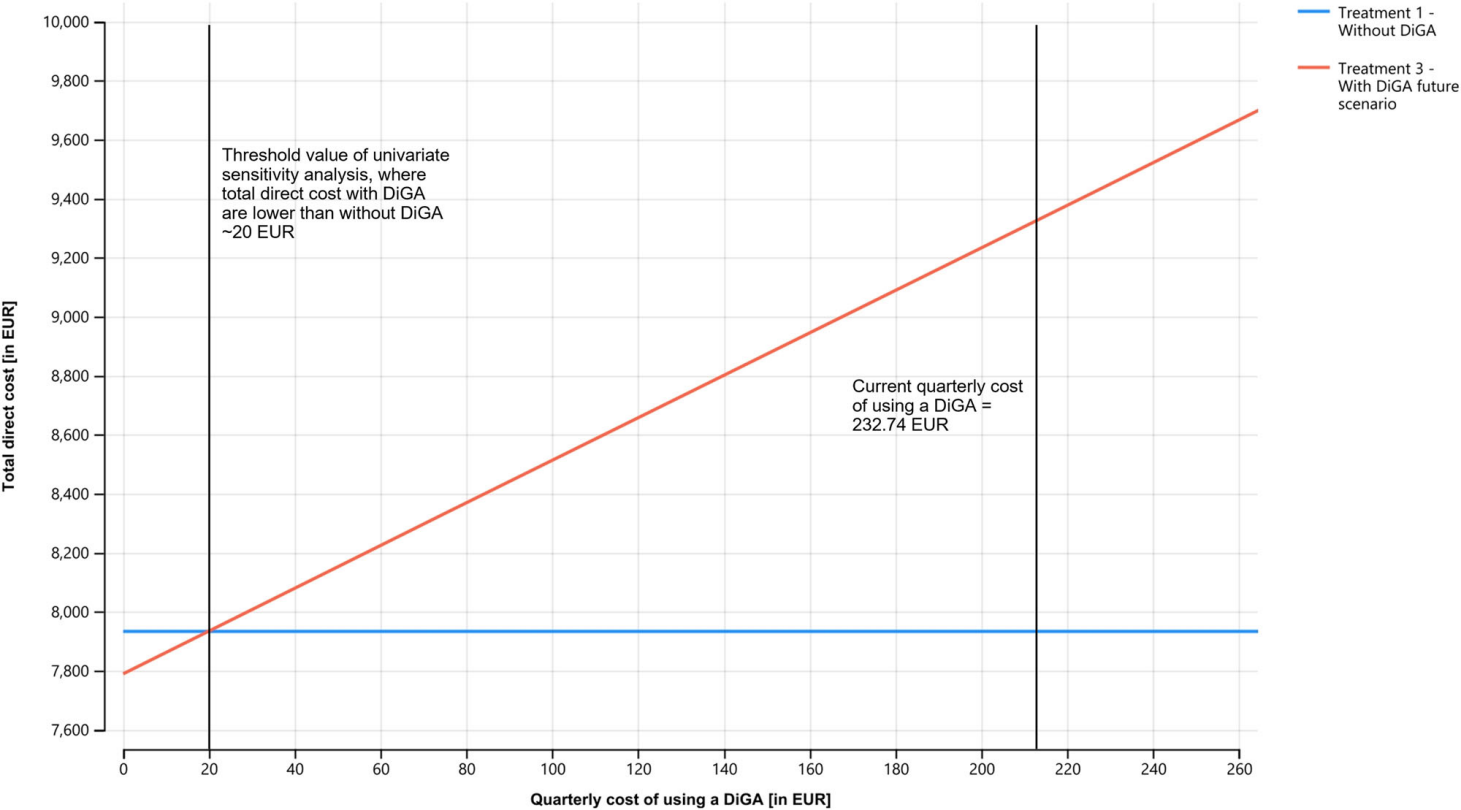
Extended univariate sensitivity analysis of input variable quarterly cost of using a DiGA. The graph shows the comparison of total direct costs of treatment scenario 1 and treatment scenario 3 depending on the quarterly cost of using a DiGA. The analysis shows that once the quarterly cost of using a DiGA fall below the threshold of ~20 EUR, the total direct costs of scenario 3 are lower compared to scenario 1. Blue line = Treatment 1 without DiGA; red line = Treatment 3 with DiGA future scenario.

*Univariate sensitivity analysis* (also called one way sensitivity analysis) was performed to assess the robustness of the base case results (see Fig. 3). We excluded treatment 2 from the sensitivity analysis for better readability because it only differs from treatment 3 in terms of the proportion of the cohort using a DiGA, resulting in identical interpretation of the results for both treatment strategies. The analysis showed that the model results were most sensitive to the following three input variables: the transition probability moving from the severe depression health state to the moderate one, the utility value for the moderate depression health state and the quarterly cost for using a DiGA. We performed an extended one-way sensitivity analysis for the quarterly cost of using a DiGA (see Fig. 4). Once the quarterly price for getting a DiGA would reach a threshold value below 20.34 EUR per quarter, both DiGA treatment scenarios would represent a cost-effective and dominant scenario.

**Fig. 5:**
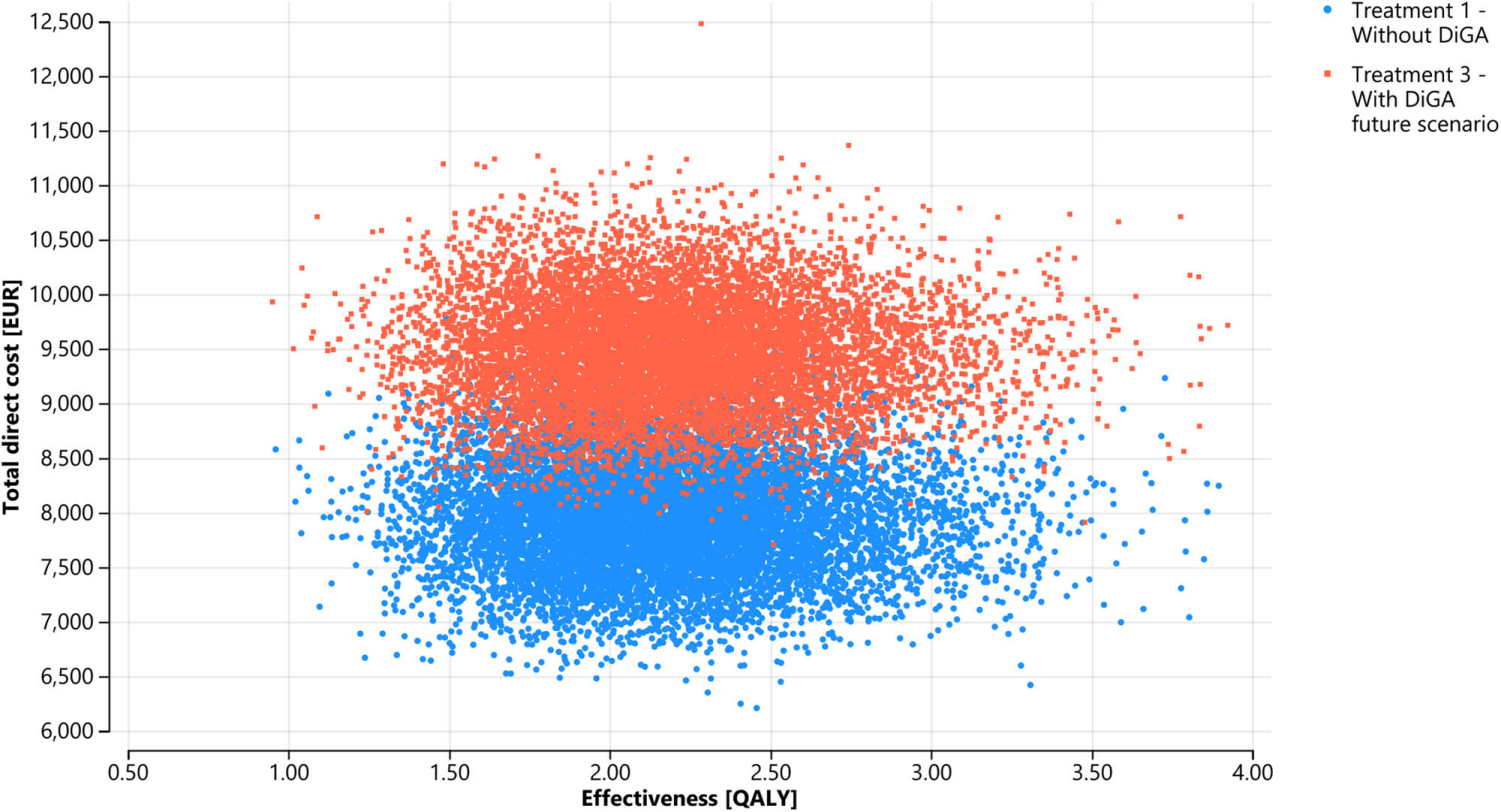
Probabilistic sensitivity analysis (PSA). Each data point marks the result of one of the 10,000 simulation runs, where input parameters were randomly drawn from defined distributions according to Table 2 to assess parameter uncertainty. DiGA treatment gained average QALYs of 2.19 (95% CI; 2.18–2.20 QALY) with mean total direct costs of  ~ 9476 EUR (95% CI; 9466–9486 EUR) per patient in the cohort; compared to the treatment without DiGA with average QALYs of 2.17 (95% CI; 2.16–2.18 QALY) with mean total direct costs of ~7936 EUR (95% CI; 7926–7946 EUR) per patient in the cohort. Blue dots = Treatment 1 without DiGA; red dots = Treatment 3 with DiGA future scenario.

*Probabilistic sensitivity analysis (PSA)* helped to determine input parameter uncertainty. Figure 5 shows the result of the PSA based on 10,000 simulation runs. DiGA treatment gained average QALYs of 2.19 (95% CI; 2.18–2.20 QALY) with mean total direct costs of 9476 EUR (95% CI; 9466–9486 EUR) per patient in the cohort; compared to care without DiGA with average QALYs of 2.17 (95% CI; 2.16–2.18 QALY) with mean total direct costs of ~ 7,936 EUR (95% CI; 7926–7946 EUR) per patient in the cohort. Of 10,000 iterations, the probability of DiGA treatment being effective in QALY gains with cost savings was 36.15% in our exemplary PSA.

## Discussion

Our cohort-based state-transition Markov model (CMM) revealed that DiGA for depression, when used on an add-on basis, with a probability of 63.85% do not directly save costs but can improve patient care and enhance public health outcomes. Inaccessible therapy for depression is a challenge, with two-thirds of depressed individuals not seeking professional help within a year due to general lack of availability and regional disparities in psychotherapy availability^42^. In contrast, DiGA offer nationwide accessibility and can potentially bridge waiting times for psychotherapy and thus help that symptoms do not deteriorate. Hence, DiGA can improve the care for underserved populations or subgroups and reduce overall morbidity^43^.

Based on our CMM, we concluded that DiGA for the depression on the one hand can help to improve the quality of life of people suffering from depression, but on the other hand are associated with noticeable extra costs and hence currently represent no dominant health strategy. These findings are in line with past research. Kolovos, S. et al.^44^ used multilevel regression analysis to assess the cost-effectiveness of internet interventions for depression (intervention was not a DiGA) concluded that they are more costly and not considered as cost-effective in comparison to the defined control group.

We further hypothesize that the cost dominance of DiGA treatment strategies is dependent on the respective indication group due to different cost structures and treatment options. Lewkowicz et al.^36^ analyzed the cost-effectiveness of a DiGA for patients with low back pain. Their base case analysis revealed additional costs of 121.59 EUR with QALY gains of 0.0221. Since they used a different time horizon in their base case analysis, we compared their model results of a 5-year time horizon scenario analysis to our results. In this scenario analysis, DiGA treatment for low back pain seemed to be a dominant treatment strategy since their model revealed cost savings of 381.80 EUR with QALY gains of 0.0534.

For our cost-effectiveness analysis we used a depression specific WTP threshold for one QALY. Germany’s health care system offers some special characteristics in this context. By law, there is an obligation for all Germans to have a health insurance and for patients with statutory health insurances, a large proportion of all healthcare services is covered by the insurances. For this reason, past studies have shown that citizens in Germany have difficulties estimating WTP threshold values for hypothetical scenarios since they are used to health insurances covering all costs without them having any transparency regarding costs^45^. Generalized, disease-independent WTP thresholds are even seen as non-compliant with the Social Security Law Book V in Germany^46,47^.

Next, our results indicate that the quarterly price for using a DiGA are one lever to build a dominant DiGA treatment scenario. While on the one hand current DiGA prices might be justifiable to cover development costs and to foster innovation in a relatively new field of medical innovation, on the other hand statutory health insurances criticize the additional cost constraint resulting from DiGA prices^48–50^. Following the cost development dynamic of pharmaceuticals over their life cycle, we expect DiGA prices to fall in the long run. Nevertheless, there is optimization potential in aligning the interests of both DiGA providers and statutory health insurances to achieve higher prescription and usage rates. Our CMM can be used by both DiGA providers and statutory health insurances to determine their individual WTP threshold value for a DiGA treating depression. Concerns raised by health insurances align with findings from a study analyzing DiGA application reviews in Germany, indicating that end users also question current pricing mechanisms^21^. Gräfe, V. et al.^51^ examined the economic potential of one specific DiGA for depression using administrative data of a statutory health insurance in Germany. The authors found that the difference of the total cost decrease of the intervention group compared to the control group was significant, although the difference of costs in the single cost categories was not significant. Additional scenario analysis revealed that if the DiGA usage cost exceeded 34 euro per patient, there would no longer be a significant difference of total cost of both treatment groups. Comparing this to our threshold value of ~20 EUR per patient and quarter, we are in a similar range. Any observed difference could be attributed to variations in the cost baseline, as we included outpatient costs in our CMM.

The difference of results of the QALY effect of treatment 1 and 2 is marginal. This effect can be explained by the current DiGA usage numbers: If we relate the approximate current usage figures for depression DiGA in Germany to the total number of patients suffering from depression and extrapolating it to our simulation horizon, the number accounts to 2% over the 5 year simulation horizon (used as input factor for treatment 2). Thus, we conclude that current DiGA usage numbers have a marginal effect on the German health care system. The Association of Statutory Health Insurance Funds in Germany (“GKV Spitzenverband”) reports a steady growth rate of total DiGA usage (200% from October 2021 to September 2022 and 68% from October 2022 to September 2023^52^). Combined with an assumed increasing and improved digital affinity among the German population, higher usage numbers can be expected in the future – which we accounted for in our treatment scenario 3. At the same time the association of providers of DiGA demands to simplify the access to DiGA for patients. They report that on average patients wait 13 days to receive a so-called DiGA activation code resulting in a delayed start of the therapy and potential further deterioration of symptoms. In addition, DiGA providers underscore the therapeutic autonomy of physicians, since they report of instances where statutory health insurances have recommended less expensive mobile health applications (instead of the described DiGA) to their policyholders that lack the approval form the Federal Institute for Drugs and Medical Devices in Germany^53^.

We further like to emphasize that current data availability and comparability is a major issue that needs to be addressed. We invested a considerable amount of time in researching and synthetizing suitable, robust, and comparable input data form various data sources. We hence argue that while it is essential to further invest in systematic evaluation procedures and methodologies in healthcare^28^, the provision of interoperable real-world data for researchers is crucial. Real-world data helps to continuously learn and evaluate the benefits, costs, chances and challenges of DiGA in everyday care to provide a sustainable and efficient resource allocation as well as strategic improvement of healthcare^17^.

Some limitations need to be considered when interpreting our results. The overall data quality of our exemplary base case scenario is one limitation. First, our input data only refers to the German healthcare system and hence lacks in international comparability. Secondly, as previously noted, we had to aggregate data from various sources with different publication years, and we opted not to apply a time correction for the sake of clarity. Thirdly, regarding the transition probabilities, to the best of our knowledge, there is only one available data source that we could utilize to derive the underlying transition probabilities for treatment 1, specifically considering various depression severity levels (“without DiGA”). Concerning the transition probabilities for treatment 2 and 3, we used several DiGA studies which all have a slightly different study set-up, e.g., the control groups are not analogously defined or the availably of guided versus unguided therapy was differently handled. For the costs, we only included direct costs, but one could argue that indirect costs, such as productivity losses through depression, and other qualitative factors, like technical features, perceived benefits from patients or physicians or process efficiency gains etc. are not considered. Furthermore, the limitations of the input data also caused a necessary simplification in the development of our CMM: We have not considered the comorbidity of depression, although past research has shown that depression is always comorbid with other mental disorders like, for example, anxiety^54^. Beyond that, for reasons of simplicity, we used discrete instead of continuous modeling of health states and transition probabilities and did not account for individual patient histories. The risk of recurrence in depression depends on the number of previous depressive episodes^55^ such that the inclusion of patients’ histories would probably better represent the real course of the disease. This comes with the practical downsides of greater model complexity and higher input data requirements.

Hence, further research could validate our developed CMM with real-world data for transition probabilities, costs, and utilities. If real world data is available, it would be of great value to also incorporate indirect cost factors due to the significant impact of productivity losses of depression patients. We expect that the cost-effectiveness of DiGA would improve if the total costs per health state were increased by adding indirect costs. If the data availability hurdle is solved in the future, an individual-based state-transition model could be built and compared to our CMM model. Furthermore, our developed model can be applied and compared to different countries. Further research could also invest in building models to assess the cost-effectiveness of mobile health applications of further diseases.

We applied a successful proof-of-concept for the application of a CMM model to simulate the cost-effectiveness of depression DiGA in Germany. Our developed CMM can be used as standard framework for decision makers to assess the cost-effectiveness of mobile health applications. Our results show that DiGA for depression in Germany are an innovative opportunity to increase the overall QALYs of a population and hence can contribute to the improvement and access of health care, but given the current cost structure in Germany, DiGA cannot yet contribute to save total health care costs.

## Methods

### Model selection and structure

Decision trees, cohort-based state-transition Markov models (CMM) or individual-based state-transition models are commonly used to develop cost-effectiveness models for depression treatments^56^. We used a CMM to evaluate the cost-effectiveness of DiGA for depression compared to the current standard of care without DiGA. Decision criteria to use a CMM were their transparency, flexibility, efficiency, ease of debugging, intuitiveness and suitability for depression^57^. Compared to decision trees, Markov models can better simulate different disease courses and hence have better clinical validity for depression^56^. While individual-based state-transition models could account for single patient characteristics and histories, we opted against them due to greater model complexity and the lack of suitable input data (CMMs require lower input data requirements)^56^. We applied the best practice guidelines of the ISPOR-SMDM Modeling Good Research Practices^57,58^ for the development of our model (see Supplementary Table 1). In addition, the CHEERS 2022 checklist for health economic evaluations was applied and can be found in Supplementary Table 2^59,60^.

CMMs are used to illustrate the progression of disease through a sequence of changes between defined health states. The model is a discrete-time model dividing the time horizon into cycles of equal length. Within CMMs, a cohort of patients move from one state to another or remain in the same state within one cycle. The transitions between the defined health states are defined by conditional, so-called transition probabilities which depend on the current health condition only. CMMs are therefore called memoryless (“Markovian property”), meaning that a future health state only depends on the current health state and not the sequence of previous ones^57,61^. Consequently, CMMs do not account for patient histories or the time a single patient spends in one health state^56^. The transition between discrete health states persists until patients reach an irreversible state like “death” or until the specified time horizon is reached. Costs and utilities are assigned to each defined health state. The total anticipated costs and utilities are calculated across the model’s overall time horizon by summarizing the duration spent in each health state and multiplying it by the corresponding costs and utility weights^55^.

To assess the cost-effectiveness of depression mobile health apps, we defined three treatment scenarios. These scenarios help gauge the value of the digital health solution by comparing its incremental advantages to the existing standard of care^31^. The first one describes the standard of care before the introduction of mobile health applications (in Germany DiGA), hereafter referred to “Treatment 1: Without DiGA”. The second one describes the current standard of care, where patients have additionally access to mobile health applications/DiGA and current usage rates are used as basis (“Treatment 2: With DiGA standard of care”). The third one describes a hypothetical future scenario with mobile health applications/DiGA, where we expect the DiGA usage rate to increase (“Treatment 3: With DiGA future scenario”). We assume that 50% of the cohort use a DiGA. Previous studies concluded an average treatment rate of affective disorders in Germany of ~50%, which we accordingly set as future potential DiGA treatment threshold in our study^62,63^.

**Fig. 6:**
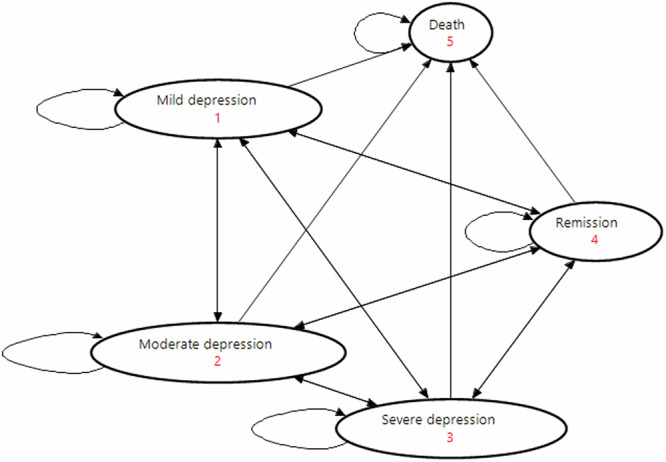
State transition diagram of the CMM model. The arrows show the possible transition paths of the patient cohort. The red numbers show the numbering of the health states. This graph shows the five modeled health states in the CMM model (mild depression, moderate depression, severe depression, remission, death).

The state transition diagram of the CMM is illustrated in Fig. 6. We differentiated between different severity levels for depression (mild, moderate, severe depression) since we need this level of detail to model the shown symptom improvements through mobile health applications. The “remission” health state refers to a period during the course of depression where the symptoms are reduced or disappear completely (recovery). We built our model in the software TreeAge Pro, version 2023. Supplementary Fig. 1a-c shows the implemented model structure in TreeAge Pro.

### Base case input data and sensitivity analysis

The base case data contains deterministic input data for the three treatment scenarios. In our example, these values are determined based on the German healthcare system. They serve as initial reference points to initiate the assessment of the cost-effectiveness of DiGA. Our model requires input data around transition probabilities, costs and utilities per health state, proportions about the starting cohort and specific CMM parameters.

To conduct cost-effectiveness analysis, we used both the incremental net monetary benefit (INMB) as well as the incremental cost-effectiveness ratio (ICER) approach according to Eq. (1) and Eq. (2). Both measures need a willingness-to-pay (WTP) threshold value as well as incremental cost and effectiveness values as input data. Incremental cost and effectiveness are calculated by comparing the DiGA treatment scenarios (Treatment 2 or 3) with a treatment without DiGA (Treatment 1).

1 $${ICER}=\frac{{Cost}\left({Treatment\; with\; DiGA}\right)-{Cost}\left({Treatment\; without\; DiGA}\right)}{{QALY}\left({Treatment\; with\; DiGA}\right)-{QALY}\left({Treatment\; without\; DiGA}\right)}=\frac{\Delta {Cost}\,}{\Delta {QALY}}$$

2 $${INMB}=\Delta {QALY}* {WTP}-\Delta {Cost}$$

A treatment scenario is considered cost-effective in case ICER < WTP or INMB > 0^64,65^. The WTP threshold value describes the amount the patient cohort is willing to pay on average for one QALY. The specific WTP threshold can vary widely within one country depending on for example the current health status, income level and level of education^45^. We base our analysis on a study of Ulbrich & Kröger^66^ that analyzed WTP threshold values for major depressive disorders in different scenarios. Within the patient sample, the mean WTP thresholds vary between 15,778 EUR and 54,794 EUR (median 13,000 EUR to 15,000 EUR). In consultation with the authors of this study, the upper value was found most appropriate as WTP threshold value for our use case, which corresponds to 1.12 times the 2023 gross domestic product in Germany per inhabitant^67^. To assess uncertainties in the deterministic input data, it is recommended to conduct univariate and probabilistic sensitivity analysis (PSA)^68–70^. For the PSA, we assigned probability distributions to input parameters based on existing research^69,71^. Usually, beta distributions are used to model the distribution for probabilities and utilities, whereas gamma distributions are used for costs^72^. We used beta distributions to model uncertainty in the transition probabilities and utility values since they are naturally bounded between 0 and 1 and are highly versatile^73^. Gamma distributions are used for costs because the distribution is defined for positive values only and can represent a wide range of positively skewed data^72^. The mean of the distributions represents the deterministic base case value, whereas the standard deviation is either derived from the original studies that were used to determine the base case value^74^ or where limited data was available we used a +/-10% range of the base case value^75^. Additionally, clinical practitioners validated the data. We tested several numbers of simulation runs, i.e., 1000; 10,000; and 100,000 runs by varying the defined input variables simultaneously with random draws from each defined distribution. For the univariate sensitivity analysis, we calculated a range for selected input variables by subtracting or adding one standard deviation from the base case value to examine the impact of selected input parameters on the ICER. Since the quarterly price for using a DiGA is a widely discussed parameter, we explicitly modeled a wider spread of this input parameter^65^.

### Exemplary input parameters

Table 2 summarizes the input parameters with exemplary numbers for the German health care system. The time horizon for the CMM was set at 5 years with each simulation cycle lasting 3 months since DiGA are usually prescribed for this period^50^. As cohort size we chose 4,977 million which represents the prevalence of depression in the German society^76,77^. For the probabilities of transitioning under “Treatment 1: Without DiGA scenario”, key insights were drawn from a study analyzing the state of care of patients with depression^78^. This study relies on data sourced from diagnosis and billing records within the German statutory health insurance system. As a limiting factor the authors calculated yearly transition probabilities, which we used for our CMM on a quarterly basis. Furthermore, the authors reported “no diagnosis” transitions, which we interpreted as our remission health state. The transition probabilities for treatment 2 and 3 are derived from the German DiGA studies employing a three-step approach. First, we analyzed the publicly available medical studies of permanently listed depression DiGA listed in the DiGA directory as of October 17, 2023. Our analysis centered on primary outcome parameters that are consistent across all selected studies, the Beck Depression Inventory (BDI and BDI-II) as well as the Patient Health Questionnaire-9 (PHQ-9). We selected the intention-to-treat data with the aim to provide a realistic insight into DiGA effectiveness while at the same time considering therapy adherence to ascertain the actual utilization of DiGA. Second, we translated the BDI/BDI-II and PHQ-9 improvement scores into the ICD-10-CM depression classification to facilitate the mapping of the depression symptom improvements to our defined health states using the assumption of normative distribution data of BDI-II and PHQ-9^79–84^. Third, we calculated the improvement of the transition probabilities, e.g. a specific percentage improvement through DiGA treatment translates in an increase of the transition probability from the current health state to the “next better” health state and a respective decrease of the current health state (e.g., 45% improvement of moderate depression translates in an increase of the transition probability from moderate to mild and a decrease of the transition probability from moderate to staying in moderate). Supplementary Table 3 summarizes the included DiGA studies. To assign cost values to the defined health states, we focused on direct cost factors only, i.e. costs for pharmaceuticals, outpatient, and inpatient costs. Costs for psychotherapy, general practitioner (GP) visits and psychiatrist visits make up the outpatient costs. Indirect costs, that are not directly related to the intervention such as productivity losses or rehabilitation costs, are not considered to narrow the scope of the analysis. To determine exemplary data for the German population depending on depression severity, we followed a bottom-up approach and aggregated the inputs from the data sources listed in Table 2. We conducted a double validation process of our exemplary cost data—on the one hand with experts from the specific field and on the other hand by means of a top-down validation with published aggregated data of the Federal Statistical Office of Germany^85^. To measure the utility of the defined health states we used health related quality adjusted life years (QALYs) operationalized by the frequently-used EQ-5D instrument^86^. A QALY is a measure in health economics that combines the quantity (time in years) and quality of life (utility value). All cost and utility values per health state were discounted^87^ and we applied half-cycle correction. Since our study was based on publicly available data, an Ethics Committee Approval was not required.

**Table 2:** Model inputs of probabilities, costs and utilities

Variable name	Description	Base-case value	Distribution (standard deviation)	Reference
**CMM parameters**				
_stage	Number of cycles simulated	20	--	Predefined value
cycle_length	Duration of one cycle	3 months	--	Predefined value
discount_rate	Discount rate for cost values	0.004	--	Deutsche Bundesbank^88^
discount_rate_QALY	Discount rate for QALYs	0.004	--	Deutsche Bundesbank^88^
p_getDiGA	Proportion of total patient cohort using a DiGA	Treatment 2: 0.0154 Treatment 3: 0.5	--	Treatment 2: GKV Spitzenverband^52^ Treatment 3: Nübel et al.^62^, Wittchen & Jacobi^63^
Cohort size	Total cohort size represents amount of people in Germany with self-reported diagnosis of depression	4,977,172	--	Grobe et al.^76^, Statistisches Bundesamt^77^
**Proportion of starting cohort**				
p_mild	Proportion of cohort starting in the mild depression health state	0.257	--	Stahmeyer et al.^78^
p_moderate	Proportion of cohort starting in the moderate depression health state	0.484	--	Stahmeyer et al.^78^
p_severe	Proportion of cohort starting in the severe depression health state	0.259	--	Stahmeyer et al.^78^
p_remis	Proportion of cohort starting in the remission health state	0.000	--	Predefined value
p_death	Proportion of cohort starting dead	0.000	--	Predefined value
**Transition probabilities**				
pwo_mild_mild	Transition probabilities of treatment 1: Without DiGA scenario (pwo) Codification according to the following scheme *pwo_a_b*, where *a* indicates the health state of the current cycle and *b* the health state in the next cycle	0.717	Calculated*	Stahmeyer et al.^78^
pwo_mild_moderate		0.055	Beta (0.006)	Stahmeyer et al.^78^
pwo_mild_severe		0.022	Beta (0.002)	Stahmeyer et al.^78^
pwo_mild_remis		0.200	Beta (0.020)	Stahmeyer et al.^78^
pwo_mild_death		0.005	Beta (0.001)	Statistisches Bundesamt^89,90^
pwo_moderate_moderate		0.774	Calculated*	Stahmeyer et al.^78^
pwo_moderate_mild		0.027	Beta (0.003)	Stahmeyer et al.^78^
pwo_moderate_severe		0.047	Beta (0.005)	Stahmeyer et al.^78^
pwo_moderate_remis		0.147	Beta (0.015)	Stahmeyer et al.^78^
pwo_moderate_death		0.005	Beta (0.001)	Statistisches Bundesamt^89,90^
pwo_severe_severe		0.787	Calculated*	Stahmeyer et al.^78^
pwo_severe_mild		0.021	Beta (0.002)	Stahmeyer et al.^78^
pwo_severe_moderate		0.091	Beta (0.009)	Stahmeyer et al.^78^
pwo_severe_remis		0.094	Beta (0.009)	Stahmeyer et al.^78^
pwo_severe_death		0.006	Beta (0.001)	Statistisches Bundesamt^89,90^
pwo_remis_remis		0.495	Calculated*	Stahmeyer et al.^78^
pwo_remis_mild		0.129	Beta (0.013)	Stahmeyer et al.^78^
pwo_remis_moderate		0.242	Beta (0.024)	Stahmeyer et al.^78^
pwo_remis_severe		0.130	Beta (0.013)	Stahmeyer et al.^78^
pwo_remis_death		0.005	Beta (0.001)	Statistisches Bundesamt^89,90^
pwo_death_death		1.000	--	
pDiGA_mild_mild	Transition probabilities of treatment 2 and 3: With DiGA scenario standard of care/future (pDiGA) Codification according to the following scheme *pDiGA_a_b*, where *a* indicates the health state of the current cycle and *b* the health state in the next cycle	0.627	Calculated*	Stahmeyer et al.^78^; Klein et al.^11^; Meyer et al.^91^; Moritz et al.^92,93^; Krämer et al.^24^; Beiwinkel et al.^94^; Baumeister & Moritz^95^ See Supplementary Table 3 for overview of included DiGA studies Distribution: Kligerman et al.^75^ ; standard deviations of included DiGA studies
pDiGA_mild_moderate		0.055	Beta (0.006)	
pDiGA_mild_severe		0.022	Beta (0.002)	
pDiGA_mild_remis		0.290	Beta (0.069)	
pDiGA_mild_death		0.005	Beta (0.001)	
pDiGA_moderate_moderate		0.763	Calculated*	
pDiGA_moderate_mild		0.038	Beta (0.012)	
pDiGA_moderate_severe		0.047	Beta (0.005)	
pDiGA_moderate_remis		0.147	Beta (0.015)	
pDiGA_moderate_death		0.005	Beta (0.001)	
pDiGA_severe_severe		0.741	Calculated*	
pDiGA_severe_mild		0.021	Beta (0.002)	
pDiGA_severe_moderate		0.137	Beta (0.031)	
pDiGA_severe_remis		0.094	Beta (0.009)	
pDiGA_severe_death		0.006	Beta (0.001)	
**Costs [in EUR]**				
c_mild	Quarterly direct costs of an average patient in mild depression health state	276.03	Gamma (27.60)	Pharmaceuticals: Bundesinstitut für Arzneimittel und Medizinprodukte^96^; Wissenschaftliches Institut der AOK^97^; IGES Institut^98^; Stahmeyer et al.^78^ Outpatient: Kassenärztliche Bundesvereinigung^99^; Stahmeyer et al.^78^; Expert judgement Inpatient: GKV Spitzenverband^100^; Statista^101^; Osterloh^102^ Overall: Bundesärztekammer et al.^82^; see Supplementary Table 3 for overview of included DiGA studies Distribution: Kligerman et al.^75^
c_moderate	Quarterly direct costs of an average patient in moderate depression health state	392.16	Gamma (39.22)	
c_severe	Quarterly direct costs of an average patient in severe depression health state	877.90	Gamma (87.79)	
c_remis	Quarterly direct costs of an average patient in remission health state	133.38	Gamma (13.34)	
c_DiGA	Quarterly cost of an average patient using a depression DiGA	232.74	Gamma (20.76)	
c_health state_disc (_stage)	Discounted cost values per health state depending on current cycle			
**Utilities**				
QALY_mild	Quarterly quality adjusted life years (QALY) of an average patient in mild depression health state	0.140	Beta (0.040)	Mohiuddin & Payne^103^
QALY_moderate	Quarterly quality adjusted life years (QALY) of an average patient in moderate depression health state	0.113	Beta (0.045)	Mohiuddin & Payne^103^
QALY_severe	Quarterly quality adjusted life years (QALY) of an average patient in severe depression health state	0.063	Beta (0.038)	Mohiuddin & Payne^103^
QALY_remis	Quarterly quality adjusted life years (QALY) of an average patient in remission health state	0.175	Beta (0.040)	Kolovos et al.^104^ Distribution: Revicki & Wood^105^
QALY_health state_disc (_stage)	Discounted quality adjusted life years (QALY) per health state depending on current cycle			

^*^Transition probability in probabilistic sensitivity analysis is calculated according to the following formula: 1-sum of all remaining transition probabilities of respective health state, e.g. pwo_mild_mild=1-pwo_mild_moderate-pwo_mild_severe-pwo_mild_remis-pwo_mild_death.

## Supplementary information

Supplementary Information

## Data Availability

The data that support the findings of this study are available from the corresponding author upon reasonable request.

## Code availability

The code that supports the findings of this study is available from the corresponding author upon reasonable request.
