# Supplementary material for: Cost-effectiveness analysis of mHealth applications for depression in Germany using a Markov cohort simulation
Source: NPJ Digit Med. 2024 Nov 17;7:321. doi: 10.1038/s41746-024-01324-0 (PMC11570631; doi:10.1038/s41746-024-01324-0)
Supplement: Supplementary file 1 — Supplementary Information [file 41746_2024_1324_MOESM1_ESM.pdf]

Supplementary Table 1: Best practice recommendations of the ISPOR-SMDM Modeling Good Research Practices Task Force-3 for State-Transition Modeling[1]

Best practice recommendations		Implementation in our study
III-1: Choice of model type	If the decision problem can be represented with a manageable number of health states that incorporate all characteristics relevant to the decision problem, including the relevant history, a cohort simulation should be chosen because of its transparency, efficiency, ease of debugging, and ability to conduct specific value of information analyses. If, however, a valid representation of any aspect of the decision problem would lead to an unmanageable number of states, then an individual-level state-transition model is recommended. Validity should not be sacrificed for simplicity.	We modeled our decision problem with five health states: mild depression, moderate depression, severe depression, remission and death.
**MODEL STRUCTURE**		
III-2: Problem statement	The strategies being evaluated should be clearly defined. In particular, sequential decisions should not be modeled within the Markov cycle tree but rather be part of the specification of the alternative intervention strategies that precede the Markov tree.	We defined three treatment scenarios:<br>Treatment 1: Without DiGA scenario; Treatment 2: With DiGA scenario standard of care and Treatment 3: With DiGA future scenario.
III-3: Starting Cohort	The starting cohort should be defined by the demographic and clinical characteristics that affect the transition probabilities or state values (e.g., quality of life and cost).	The starting cohort was derived from the current prevalence of depression in Germany.
III-4: Defining states	Specification of states and transitions should generally reflect the biological/theoretical understanding of the disease or condition being modeled.	We built on existing research to derive health states and transition probabilities.
III-5: Intervention effects	States should adequately capture the type of intervention (i.e., prevention, screening, diagnostics, treatment) as well as the intervention's benefits and harms.	DiGA focus on the treatment of depression. After symptoms have improved, DiGA have also a preventive effect against a relapse.
III-6: Heterogeneity	States need to be homogeneous with respect to both observed and unobserved (i.e., not known by the decision maker) characteristics that affect transition probabilities.	NA
III-7: Time horizon	The time horizon for the model should be sufficiently large to capture all health effects and costs relevant to the decision problem.	We chose a 5-year simulation horizon.
III-8: Cycle length	Cycle length should be short enough to represent the frequency of clinical events and interventions.	We chose a cycle length of 3 months since the digital health applications in scope (DiGA) are usually prescribed on a quarterly basis in

		Germany.
III-9: Model symmetry	Components of state-transition models that reflect similar clinical courses should not be recreated but rather should be incorporated once and linked to that structure throughout the model.	The model structure is provided in the supplementary material.
**DATA**		
III-10: Data sources	Transition probabilities and intervention effects should be derived from the most representative data sources for the decision problem.	Description can be found in the manuscript including an overview table of all input variables.
III-11: Parameter derivation	All methods and assumptions used to derive transition probabilities and intervention effects should be described.	Description can be found in the manuscript including an overview table of all input variables.
III-12: Intervention effects	All parameters relating to the effectiveness of interventions derived from observational studies should be correctly controlled for confounding. Time-varying confounding is of particular concern in estimating intervention effects.	NA
III-13: State valuation	The valuation of intermediate outcomes/states should be justified.	Description can be found in the manuscript including an overview table of all input variables.
**ANALYSIS**		
III-14: Half-cycle correction	A half-cycle correction should be applied to costs and effectiveness in the first cycle and in the final cycle if not using a lifetime horizon.	We applied half-cycle correction to costs and QALYs.
III-15: Analyzing distributions	For certain decision problems, it may be important to report not only the expected value but also the distribution of the outcomes of interest.	Exemplary distributions for selected input variables were provided and analysed.
III-16: Performing microsimulation	The number of individuals simulated should be large enough to generate stable estimates of the expected values.	Our simulation cohort accounts for 4,977 million people.
**COMMUNICATING RESULTS**		
III-17: Presenting the model	The report should use nontechnical language and clear figures and tables that enhance understanding of the STM to communicate its key structural elements, assumptions, and parameters.	Results are described in the manuscript.
III-18: Presenting results	In addition to final outcomes, intermediate outcomes that enhance the understanding and transparency of the model results should also be presented.	Results are described in the manuscript.

NA= Not applicable

Supplementary Table 2: CHEERS 2022 Checklist[2]

Item		Guidance for Reporting	Reported in section
**TITLE**			
Title	1	Identify the study as an economic evaluation and specify the interventions being compared.	See Page 1
**ABSTRACT**			
Abstract	2	Provide a structured summary that highlights context, key methods, results and alternative analyses.	See Page 1
**INTRODUCTION**			
Background and objectives	3	Give the context for the study, the study question and its practical relevance for decision making in policy or practice.	"Introduction"
**METHODS**			
Health economic analysis plan	4	Indicate whether a health economic analysis plan was developed and where available.	NA
Study population	5	Describe characteristics of the study population (such as age range, demographics, socioeconomic, or clinical characteristics).	"Exemplary input parameters"
Setting and location	6	Provide relevant contextual information that may influence findings.	"Model selection and structure", "Base case input data and sensitivity analysis", "Exemplary input parameters"
Comparators	7	Describe the interventions or strategies being compared and why chosen.	"Model selection and structure"
Perspective	8	State the perspective(s) adopted by the study and why chosen.	"Model selection and structure", "Base case input data and sensitivity analysis"
Time horizon	9	State the time horizon for the study and why appropriate.	"Exemplary input parameters"
Discount rate	10	Report the discount rate(s) and reason chosen.	Table 2
Selection of outcomes	11	Describe what outcomes were used as the measure(s) of benefit(s) and harm(s).	"Base case input data and sensitivity analysis", "Exemplary input parameters"

Measurement of outcomes	12	Describe how outcomes used to capture benefit(s) and harm(s) were measured.	“Base case input data and sensitivity analysis”, “Exemplary input parameters”
Valuation of outcomes	13	Describe the population and methods used to measure and value outcomes.	NA
Measurement and valuation of resources and costs	14	Describe how costs were valued.	“Exemplary input parameters” + Table 2
Currency, price date, and conversion	15	Report the dates of the estimated resource quantities and unit costs, plus the currency and year of conversion.	Table 2
Rationale and description of model	16	If modelling is used, describe in detail and why used. Report if the model is publicly available and where it can be accessed.	“Model selection and structure”, “Base case input data and sensitivity analysis”
Analytics and assumptions	17	Describe any methods for analysing or statistically transforming data, any extrapolation methods, and approaches for validating any model used.	“Model selection and structure”, “Base case input data and sensitivity analysis”, “Exemplary input parameters”
Characterizing heterogeneity	18	Describe any methods used for estimating how the results of the study vary for sub-groups.	NA
Characterizing distributional effects	19	Describe how impacts are distributed across different individuals or adjustments made to reflect priority populations.	NA
Characterizing uncertainty	20	Describe methods to characterize any sources of uncertainty in the analysis.	“Base case input data and sensitivity analysis”
Approach to engagement with patients and others affected by the study	21	Describe any approaches to engage patients or service recipients, the general public, communities, or stakeholders (e.g., clinicians or payers) in the design of the study.	NA
**RESULTS**			
Study parameters	22	Report all analytic inputs (e.g., values, ranges, references) including uncertainty or distributional assumptions.	Table 2
Summary of main results	23	Report the mean values for the main categories of costs and outcomes of interest and summarise them in the most appropriate overall measure.	Table 1

Effect of uncertainty	24	Describe how uncertainty about analytic judgments, inputs, or projections affect findings. Report the effect of choice of discount rate and time horizon, if applicable.	“Sensitivity analysis”
Effect of engagement with patients and others affected by the study	25	Report on any difference patient/service recipient, general public, community, or stakeholder involvement made to the approach or findings of the study	“Base case input data and sensitivity analysis”, “Exemplary input parameters”
**DISCUSSION**			
Study findings, limitations, generalizability, and current knowledge	26	Report key findings, limitations, ethical or equity considerations not captured, and how these could impact patients, policy, or practice.	“Discussion”
**OTHER RELEVANT INFORMATION**			
Source of funding	27	Describe how the study was funded and any role of the funder in the identification, design, conduct, and reporting of the analysis	“Acknowledgements”
Conflicts of interest	28	Report authors conflicts of interest according to journal or International Committee of Medical Journal Editors requirements.	“Competing interests”

NA= Not applicable

The checklist is Open Access distributed in accordance with the terms of the Creative Commons Attribution (CC BY 4.0) license, which permits others to distribute, remix, adapt and build upon this work, for commercial use, provided the original work is properly cited. See: http://creativecommons.org/licenses/by/4.0/.

Supplementary Figure 1: TreeAge Pro model structure

The TreeAge Pro model structure shows the modelling and implementation of the cohort-based state-transition Markov model in TreeAge Pro. **a** shows the branch for “Treatment 1 – Without DiGA”, **b** shows the branch “Treatment 2 – With DiGA standard of care” and **c** shows the branch for “Treatment 3 – With DiGA future scenario”. The model structure shows possible patients’ journeys moving between the defined health states (mild depression, moderate depression, severe depression, remission, death). Purple cycle with white M in the middle = Markov tree node; green circle = chance tree node; red triangle = terminal tree node; # = used for one of the branch probabilities, which is calculated as the complement of the sum of all other probability expressions of that node; Init = Initial; Incr = Incremental; for detailed variable description see Table 2 in the manuscript.

a

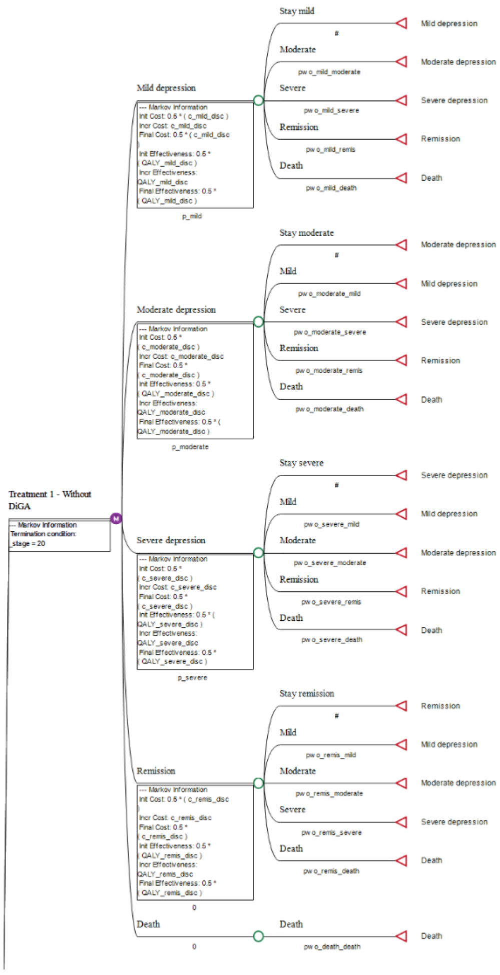

b

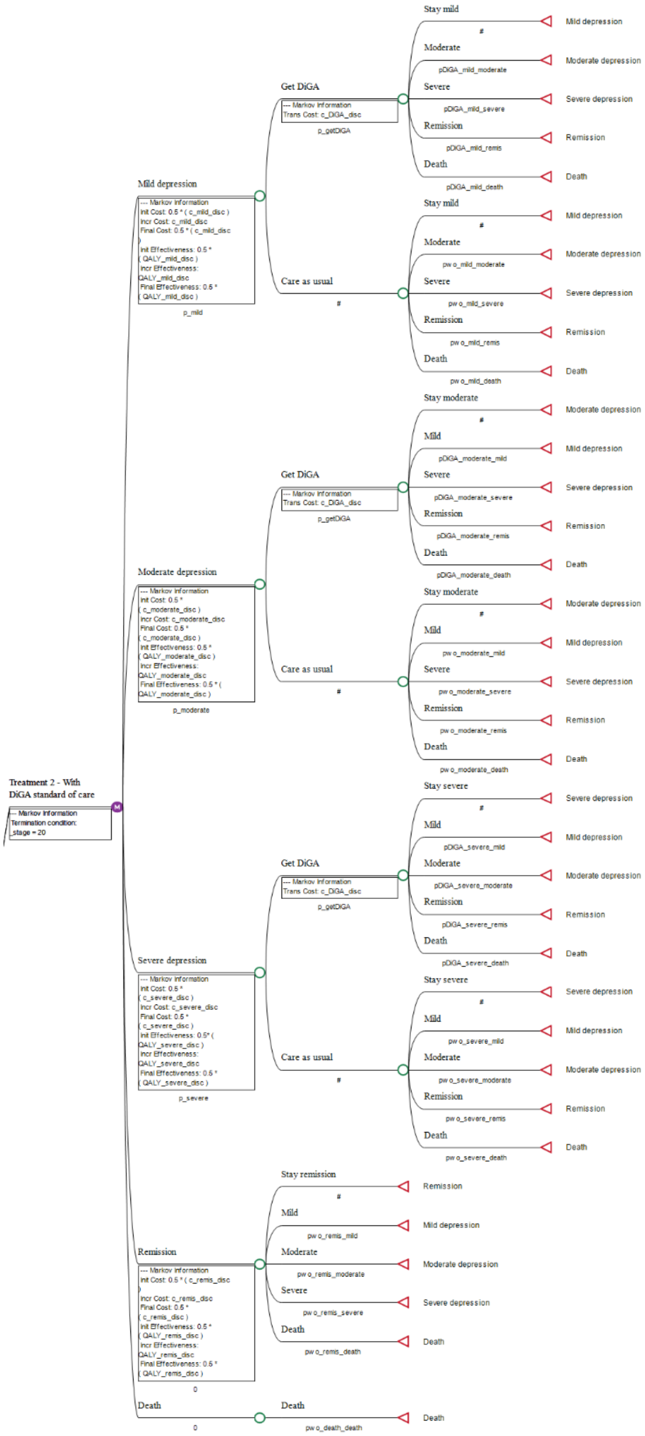
Treatment 2 - With DiGA standard of care
--- Markov information Termination condition: _stage = 20
Mild depression
p_mild
Moderate depression
p_moderate
Severe depression
p_severe
Remission
Death
Get DiGA
--- Markov information Trans Cost: c_DiGA_disc
p_getDiGA
Care as usual
Stay mild
Stay moderate
Stay severe
Stay remission
Moderate
Severe
Mild
Remission
Death
Mild depression
Moderate depression
Severe depression

c

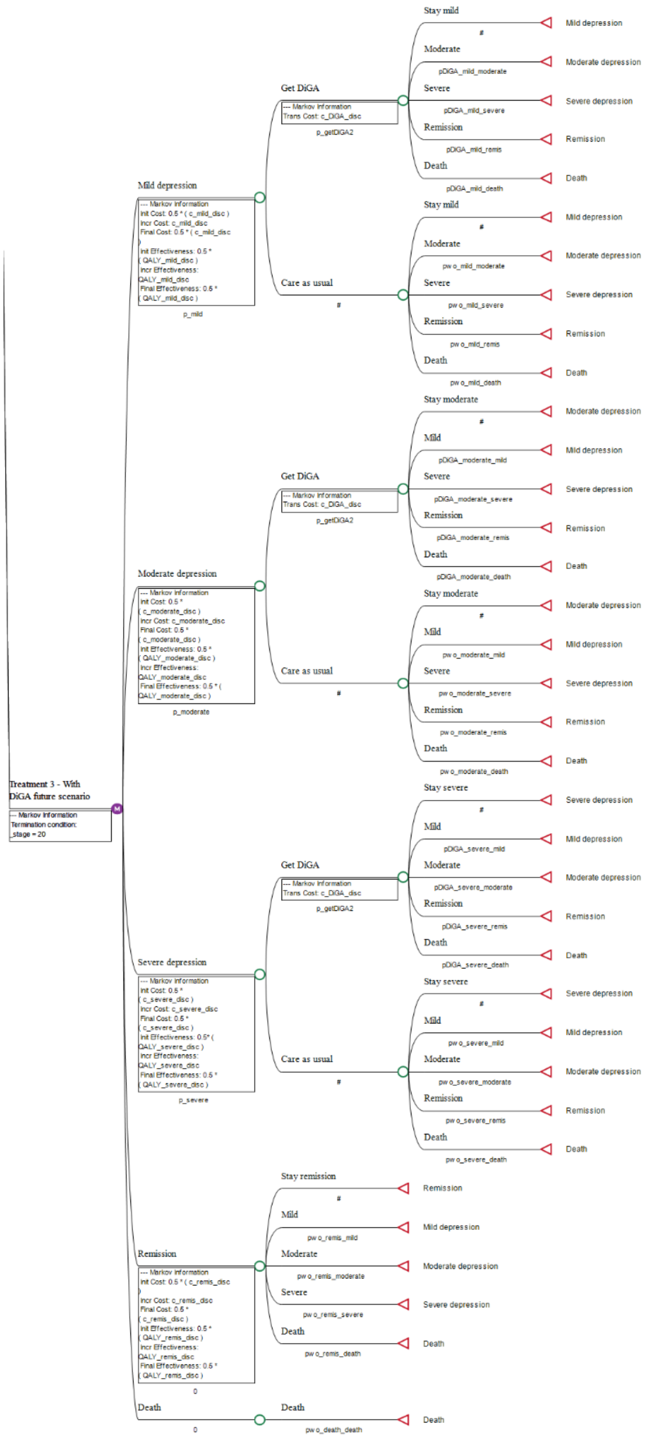

Treatment 3 - With DiGA future scenario
--- Markov information
Termination condition:
_stage = 20
Mild depression
--- Markov information
Init Cost: 0.5 * (c_mild_disc)
Incr Cost: c_mild_disc
Final Cost: 0.5 * (c_mild_disc)
Init Effectiveness: 0.5 * (QALY_mild_disc)
Incr Effectiveness: QALY_mild_disc
Final Effectiveness: 0.5 * (QALY_mild_disc)
p_mild
Get DiGA
--- Markov information
Trans Cost: c_DiGA_disc
p_getDiGA2
Stay mild
#
Mild depression
Moderate
pDiGA_mild_moderate
Moderate depression
Severe
pDiGA_mild_severe
Severe depression
Remission
pDiGA_mild_remis
Remission
Death
pDiGA_mild_death
Death
Care as usual
#
Stay mild
#
Mild depression
Moderate
pw o_mild_moderate
Moderate depression
Severe
pw o_mild_severe
Severe depression
Remission
pw o_mild_remis
Remission
Death
pw o_mild_death
Death
Moderate depression
--- Markov information
Init Cost: 0.5 * (c_moderate_disc)
Incr Cost: c_moderate_disc
Final Cost: 0.5 * (c_moderate_disc)
Init Effectiveness: 0.5 * (QALY_moderate_disc)
Incr Effectiveness: QALY_moderate_disc
Final Effectiveness: 0.5 * (QALY_moderate_disc)
p_moderate
Get DiGA
--- Markov information
Trans Cost: c_DiGA_disc
p_getDiGA2
Stay moderate
#
Moderate depression
Mild
pDiGA_moderate_mild
Mild depression
Severe
pDiGA_moderate_severe
Severe depression
Remission
pDiGA_moderate_remis
Remission
Death
pDiGA_moderate_death
Death
Care as usual
#
Stay moderate
#
Moderate depression
Mild
pw o_moderate_mild
Mild depression
Severe
pw o_moderate_severe
Severe depression
Remission
pw o_moderate_remis
Remission
Death
pw o_moderate_death
Death
Severe depression
--- Markov information
Init Cost: 0.5 * (c_severe_disc)
Incr Cost: c_severe_disc
Final Cost: 0.5 * (c_severe_disc)
Init Effectiveness: 0.5* (QALY_severe_disc)
Incr Effectiveness: QALY_severe_disc
Final Effectiveness: 0.5 * (QALY_severe_disc)
p_severe
Get DiGA
--- Markov information
Trans Cost: c_DiGA_disc
p_getDiGA2
Stay severe
#
Severe depression
Mild
pDiGA_severe_mild
Mild depression
Moderate
pDiGA_severe_moderate
Moderate depression
Remission
pDiGA_severe_remis
Remission
Death
pDiGA_severe_death
Death
Care as usual
#
Stay severe
#
Severe depression
Mild
pw o_severe_mild
Mild depression
Moderate
pw o_severe_moderate
Moderate depression
Remission
pw o_severe_remis
Remission
Death
pw o_severe_death
Death
Remission
--- Markov information
Init Cost: 0.5 * (c_remis_disc)
Incr Cost: c_remis_disc
Final Cost: 0.5 * (c_remis_disc)
Init Effectiveness: 0.5 * (QALY_remis_disc)
Incr Effectiveness: QALY_remis_disc
Final Effectiveness: 0.5 * (QALY_remis_disc)
0
Stay remission
#
Remission
Mild
pw o_remis_mild
Mild depression
Moderate
pw o_remis_moderate
Moderate depression
Severe
pw o_remis_severe
Severe depression
Death
pw o_remis_death
Death
Death
0
Death
pw o_death_death
Death

Supplementary Table 3: Overview of included DiGA studies

DiGA studies permanently accepted in DiGA registry
Indidation in scope: Depression
Last updated: 17.10.2023

**DiGA information**			**Medical study (positive care effect)[1]**						**Outcomes**					**Conclusion for CMM**	**DiGA cost**
**DiGA name**	**Company name**	**Approved indications**	**Authors names**	**Publication year**	**Paper title**	**Journal**	**DOI**	**Study type**	**Primary outcome measures**	**Primary outcome measure improvement through DiGA [absolute[2]]**	**Primary outcome measure improvement through DiGA [%]**	**Time measure period of intervention**	**Secondary outcome measures**	**Calculated average transition probability improvement [%]**	**Quarterly cost for using a DiGA [EUR]**
Deprexis	GAIA AG	F32.0<br>F32.1<br>F32.2<br>F33.0<br>F33.1<br>F33.2	Jan Philipp Klein; Thomas Berger; Johanna Schröder; Christina Späth; Björn Meyer; Franz Caspar; Wolfgang Lutz ; Alice Arndt; Wolfgang Greiner; Viola Gräfe; Martin Hautzinger; Kristina Fuhr; Matthias Rose; Sandra Nolte; Bernd Löwe; Gerhard Andersson; Eik Vettorazzi; Steffen Moritz; Fritz Hohagen	2016	Effects of a Psychological Internet Intervention in the Treatment of Mild to Moderate Depressive Symptoms: Results of the EVIDENT Study, a Randomized Controlled Trial	Psychotherapy and Psychosomatics	10.1159/000445355	Randomized controlled trial (RCT)	PHQ-9 (patient health questionnaire)	1,61	-18%	3 months	GAD-7 (generalized anxiety disorder)<br>PHQ-15 (patient health questionnaire - 15 items)<br>SF-12 (short form health survey-12)<br>ZUF-8 (patient satisfaction questionnaire)<br>HAQ-11 (helping alliance questionnaire)	-18%	210,00 €
			Björn Meyer; Julia Bierbrodt; Johanna Schröder; Thomas Berger; Christopher G. Beevers; MarioWeiss; Gitta Jacob; Christina Späthg; Gerhard Andersson; Wolfgang Lutz; Martin Hautzinger; Bernd Löwe; Matthias Rose; Fritz Hohagen; Franz Caspar; Wolfgang Greiner; Steffen Moritz; Jan Philipp Klein	2014	Effects of an Internet intervention (Deprexis) on severe depression symptoms: Randomized controlled trial	Internet Interventions	http://dx.doi.org/10.1016/j.invent.2014.12.003	Randomized controlled trial (RCT)	PHQ-9 (patient health questionnaire)	3,56	-26%	3 months	GAD-7 (generalized anxiety disorder)<br>PHQ-15 (patient health questionnaire - 15 items)<br>SF-12 - physical (short form health survey - 12)<br>SF-12 - mental (short form health survey - 12)	-26%	
			Steffen Moritz; Lisa Schilling; Marit Hauschildt; Johanna Schröder; András Treszl	2012	A randomized controlled trial of internet-based therapy in depression	Behaviour Research and Therapy	https://doi.org/10.1016/j.brat.2012.04.006	Randomized controlled trial (RCT)	BDI (Beck depression inventory)	5,16	-20%	8 months	DAS (Dysfunctional attitude scale)<br>RSE (Rosenberg Self-Esteem Scale)<br>SBQ-R (Suicide Behaviors Questionnaire-Revised)<br>WHO Quality of Life (WHOQOL-BREF)<br>Subjective benefit	-20%	
Selfapy Depression	Selfapy GmbH	F32.0<br>F32.1<br>F33.0<br>F33.1	Rico Krämer; Lea Köhne-Volland; Anna Schumacher; Stephan Köhler	2022	Efficacy of a Web-Based Intervention for Depressive Disorders: Three-Arm Randomized Controlled Trial Comparing Guided and Unguided Self-Help With Waitlist Control	JMIR Formative Research	10.2196/34330	Randomized controlled trial (RCT)	BDI-II (Beck Depression Inventory-II)[3]	11,77	-39%	3 months	BAI: Beck Anxiety Inventory	-39%	217,18 €

Novego Depression	IVP Networks GmbH	F32.0<br>F32.1<br>F32.2<br>F33.0<br>F33.1<br>F33.2<br>F34.1	Till Beiwinkel; Tabea Eißing; Nils-Torge Telle; Elisabeth Siegmund-Schultze; Wulf Rössler	2017	Effectiveness of a Web-Based Intervention in Reducing Depression and Sickness Absence: Randomized Controlled Trial	Journal of Medical Internet Research	10.2196/jmir.6546	Randomized controlled trial (RCT)	PHQ-9 (Patient Health Questionnaire)	1,25	-16%	0 months	MANSA (Manchester Short Assessment of Quality of Life)	-16%	249,00 €
									BDI II (Beck Depression Inventory-II)	1,97	-13%	0 months		-13%	
			Steffen Moritz; Johanna Schröder; Jan Philipp Klein; Tania M. Lincoln; Christina Andreou; Anja Fischer; Sönke Arlt	2016	Effects of online intervention for depression on mood and positive symptoms in schizophrenia	Schizophrenia Research	http://dx.doi.org/10.1016/j.schres.2016.04.033	Randomized controlled trial (RCT)	PHQ-9 (Patient Health Questionnaire)[4,5]	3,7	-32%	0 months	Paranoia Checklist	-32%	
			Franziska Miegel; Josefine Gehlenborg; Lara Bücker; Despina Lion; Steffen Moritz	2019	Can an Online Intervention for Depression Alleviate Emotional Problems and Pain? A Randomized Controlled Study	Verhaltens-therapie	10.1159/000501736	Randomized controlled trial (RCT)	BDI-II (Beck Depression Inventory-II)[4]	5,2	-25%	0 months	VAS (Visuelle Analogskala)<br>DSF (Deutscher Schmerzfragebogen)<br>URICA (University of Rhode Island Change Assessment Scale)<br>CEQ (Credibility/Expectancy Questionnaire)<br>ZUF-8 (Fragebogen zur Messung der Patientenzufriedenheit)<br>Subjective evaluations	-25%	
									PHQ-9 (Patient Health Questionnaire)[4,5]	1,86	-17%	0 months		-17%	
			Anna Baumeister; Steffen Moritz	2023	Studienbericht Novego Depression	n/a	n/a	Randomized controlled trial (RCT)	BDI-II (Beck Depression Inventory-II)[6]	1,577	-7%	3 months	RSES (Rosenberg Self-Esteem Scale)<br>WHO-WOL-BREF (World Health Organization Quality of Life - BREF)	-7%	
									PHQ-9 (Patient Health Questionnaire)[5,6]	0,755	-7%	3 months		-7%	
**Average PHQ-9**										**2,12**				**-19%**	**225,39 €**
**Average BDI / BDI-II**										**5,14**				**-21%**	

1 Listed by the manufacturer and publicly available, intention to treat sample data (if available)
2 Calculated as absolute difference of outcome measure of control group versus intervention/DiGA group
3 Unguided group (without additional psychotheraphy)
4 Outcome measures read from graphs in the publication, per protocol data
5 Secondary outcome but listed for reasons of comparability between the studies
6 Mixed effects model for repeated measures

Limitation: Different time measure periods after intervention and different design of control group

Supplementary material references

1. Siebert, U. *et al.* State-transition modeling: a report of the ISPOR-SMDM Modeling Good Research Practices Task Force--3. *Value Health* **15,** 812–820 (2012).

2. Husereau, D. *et al.* Consolidated Health Economic Evaluation Reporting Standards 2022 (CHEERS 2022) Statement: Updated Reporting Guidance for Health Economic Evaluations. *Value Health* **25,** 3–9 (2022).
